# Treating hypertensive patients with coronary artery disease: renewed interest in INVEST

**Published:** 2010-02

## Introduction

The INVEST study, a very large study of 22 576 hypertensive patients with coronary artery disease (CAD)^1^ has recently been reviewed in the context of more recent studies, such as ASCOT2 and ACCOMPLISH,3 all of which focused on the use of a calcium channel blocker-led (CBB) strategy in combination with an ACE inhibitor to reduce cardiovascular events.

The Cardiovascular Journal of South Africa, precursor to the present Cardiovascular Journal of Africa, published expert comment on the results of the INVEST trial in 2003 and reported on the follow-up series of meetings held in South Africa after the 2003 American College of Cardiology (ACC) meeting at which the INVEST trial results were presented.^4^ Particularly important for our diverse society is that this trial included 13% black patients, and 52% of the patients were female.

Essential to the understanding of the INVEST study is the context in which it was initiated in the mid-nineties; mainly to address unanswered management issues in patients with CAD. At that time, diuretics and β-blockers were the standard blood pressure-lowering therapy, although they had not been shown to reduce morbidity and mortality to the levels predicted from epidemiological studies. The newer agents such as CCBs and ACE inhibitors were increasingly being used, although outcome data were lacking at the time.

**Fig. 1.**
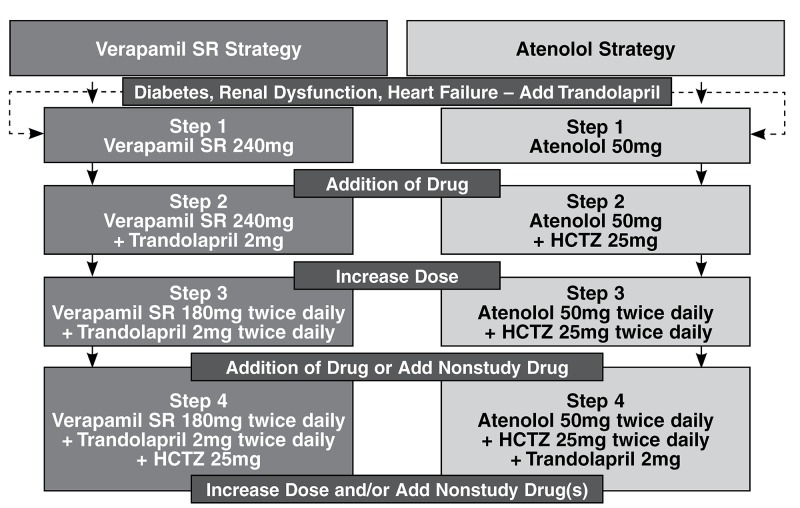
INVEST treatment strategies. The drugs, order of addition and recommended doses for each step of each strategy are summarised. Non-study antihypertensive drugs could be added to control blood pressure except for β-blockers in those assigned to the atenolol arm.

The INVEST study focused on hypertensive patients with CAD who were older than 50 years. It anticipated that very few patients would achieve target blood pressure on monotherapy and opted to test the combined use of verapamil SR 240 mg/ day and trandolapril 4 mg/day against atenolol (twice daily) and hydrochlorothiazide (HCTZ) (Fig. 1). Patients were monitored every six weeks for the first six months, and then every six months until the study end (mean follow-up period 2.9 years).

The overall objective of INVEST was to compare the risk for the primary outcome (PO), defined as all-cause death, non-fatal myocardial infarction (MI) or non-fatal stroke, following treatment with the two strategies. Secondary outcomes included not only all-cause death, nonfatal MI and non-fatal stroke individually, but also new-onset diabetes and trends for cancer, Parkinson’s, Alzheimer’s and autoimmune disease and gastrointestinal bleeding, since these had all been anecdotally attributed to long-term use of calcium antagonists.

Depression is common in CAD patients and is an important risk factor for subsequent coronary heart disease (CHD) events.^5^ Because the use of β-blockers may be associated with generalised fatigue and depression, the substudy Antihypertensive Drugs and Depression Symptoms (SADD-Sx)^6^ was carried out to examine the tolerability of the two strategies and to assess for depression at baseline and after one year of treatment. For the substudy, 2 317 consecutively randomised INVEST patients in the USA were mailed questionnaires, including a sociodemographic survey at baseline and the Centre for Epidemiologic Studies – Depression (CES-D) scale at baseline and after one year of study participation.

Another ongoing substudy included ambulatory blood pressure monitoring (ABPM), in which a portion of the INVEST population underwent ABPM at baseline and after one year of follow up. Analysis from this substudy is underway and publications will be forthcoming.

Overall, the primary outcome of INVEST was not statistically significantly different between the two treatment arms; although new-onset diabetes was lower in the verapamil SR/trandolapril arm than in the atenolol/HTCZ arm. In fact, patients in the verapamil plus trandolapril- based group were 15% less likely to develop new-onset diabetes during follow up. Blood pressure lowering was almost identical in both treatment strategies, with more than 70% of patients reaching target blood pressure of less than 140/90 mmHg.

Angina episodes were reduced by some 50%, with fewer angina episodes being experienced with the verapamil SR/trandolapril strategy. Interestingly, patients with diabetes were taking on average three antihypertensive medications. Depression improved significantly in the verapamil-treated group, as did quality of life. Both treatment strategies were well tolerated.

The major contrast between INVEST and the two later studies, ASCOT and LIFE, is that atenolol was dosed twice daily, resulting in the more equivalent outcomes in INVEST.

This expert review of INVEST concludes that the selection of antihypertensive agents should be based on patients co-morbidities and other risks, importantly the risk of developing diabetes. Key findings are summarised in Table 1.

**Table 1:** KEY FINDINGS OF INVEST

A verapamil SR-plus-trandolapril strategy was equivalent to an atenolol (twicedaily)-plus-hydrochlorothiazide strategy with regard to reduction in cardiovascular outcomes, with similar blood pressure reduction and control The verapamil SR plus trandolapril strategy was associated with a reduced risk for new-onset diabetes Elderly patients with hypertension and CAD require multi-drug therapy for blood pressure control. Lean, elderly patients should be treated carefully andblood pressure should not be lowered too far in this population Blood pressure reduction and reduction in angina episodes were associated with an improved feeling of well being Atenolol, when dosed twice daily, was not associated with increased risk of stroke or other adverse cardiovascular outcomes
